# No Difference in the Acute Effects of Randomization vs. Blocking of Units of Lower-Extremity Proprioceptive Training on Balance and Postural Control in Young Healthy Male Adults

**DOI:** 10.3389/fphys.2022.824651

**Published:** 2022-04-26

**Authors:** Patrik Ivusza, Tibor Hortobágyi, Balázs Sebesi, Balázs Gáspár, Ádám Fésüs, Mátyás Varga, Vanessza Malmos, Márk Váczi

**Affiliations:** ^1^ Institution of Sport Sciences and Physical Education, Faculty of Sciences, University of Pécs, Pécs, Hungary; ^2^ Center for Human Movement Sciences, University Medical Center, University of Groningen, Groningen, Netherlands; ^3^ Faculty of Humanities, Institute of English Studies, University of Pécs, Pécs, Hungary

**Keywords:** differential learning, unilateral landing, tilt surface, postural sway, postural adjustment

## Abstract

Random practice is a form of differential learning and its favorable acute effects on motor performance are well described when visual tasks are practiced. However, no study to date has investigated the acute effects of differential learning using variable proprioceptive stimuli instead of the visual cues. The aim of the present study was to compare the acute effects of randomized versus blocked lower-extremity proprioceptive training stimuli on balance and postural adjustments. In two conditions, healthy young males (*n* = 15, age = 23 years) performed 16 one-legged landings on a board tilted in four directions: 1) tilt direction unknown and randomized and 2) tilt direction known with order of presentation blocked. Multi-segmental angular sway while balancing on an unstable surface and postural responses to perturbation stimulus by surface tilts were measured before and 4 min after training. Overall frontal-plane postural sway on the unstable surface decreased (*p* < 0.05, η^2^ = 0.022) in both conditions, while sagittal-plane postural sway remained unchanged. When the surface was toes-up tilted in the perturbation test, the sagittal-plane shank-thigh-pelvis alignment improved in both conditions (*p* < 0.05, η^2^ = 0.017), but the direction of the segmental positioning was non-uniform across participants. We conclude that randomization vs. blocking of units of lower-extremity proprioceptive training did not affect balance and postural control in our cohort of healthy young adults but the improvements were test-specific.

## Introduction

Differential learning (DL), also known as variability of practice, facilitates motor learning (Torrents et al., 2007; Kovaleski et al., 2009; James, 2014; Gaspar et al., 2019). Instead of repeating the same movement, DL involves the practice of a variety of movements and introduces “training noise”. Variation in movement patterns during practice facilitates the acquisition of a new motor skill evidenced by reductions in movement error.

Random practice is a version of DL (Schmidt and Lee, 1999). During random practice, practitioners perform task variations in a random order. For example, balance exercises are executed with different postures (James, 2014) or the same task is performed at varying levels of difficulty in a random order (Kovaleski et al., 2009). Still another version of random practice is when the performer receives a variety of unexpected stimuli in a random order as is the case during perturbed gait (Nachmani et al., 2021). In intervention studies that examine the effects of practice modalities on motor learning, random practice is often compared with a blocked practice, in which the same stimuli are repeated in blocks from trial to trial (Jeon et al., 2021; Porter and Magill, 2010).

When individuals practice using task variations, contextual interference can occur (Magill and Hall, 1990). Random practice is associated with high contextual interference and forces practitioners to rely on elaborative and distinctive conceptual processing of the practice task compared with blocked practice (elaboration hypothesis). After random vs. blocked practice, learning benefits arise from the preparative and evaluative processes being stronger and longer lasting (Schmidt and Lee, 1999). According to the forgetting-reconstruction hypothesis, the learner forgets after each task in order to focus on and reconstruct the action plan for the next task, resulting in stronger memory representation of the practiced task (Lee and Magill, 1985). The retroactive inhibition hypothesis suggests that contextual interference is related to disadvantages of blocked practice rather than the advantages of random practice (Thürer et al., 2019) (Shewokis et al., 1998).

The time scale of such effects has not been fully characterized yet, as there are scant data on the acute effects of random vs. blocked practice on motor execution. For example, when young adults were instructed to putt a golf ball to target over different distances, the immediate post-acquisition putting accuracy increased equally after blocked and random schedules (Porter and Magill, 2010). In contrast, when healthy adults performed a single session of standing balance training either with variable or constant postural positions, variable executions improved balance sway (James, 2014). This suggests that perhaps motor skill acquisition is faster when practice relies on proprioceptive instead of visual processing.

Another limitation of studies exploring the adaptation mechanism (either long-term or acute) after random vs. blocked practice interventions is that researchers used either visual keys as training stimulus, i.e., participants receive a variety of visual signals according to which they perform different movements (visuo-motor processing of a known task) (Porter and Magill, 2010) or participants receive instructions to perform a movement with kinematic variations (proprioceptive processing of a known task) (Jeon et al., 2021), and no study to date have investigated the acute effects of random practice using solely unexpected proprioceptive stimuli (proprioceptive processing of an unknown/unexpected task) instead of the visual cues. A single session of balance training involving difficulty variations can improve balance performance (Muehlbauer et al., 2021), suggesting rapid neuromuscular adjustments to proprioceptive stimuli. However, it remains unclear whether organizing these stimuli either into a randomized (unpredicted sequence) or a blocked schedule would affect learning. There are several studies in support of the above hypothesis: First, exercising on unstable vs. stable surfaces already increased electromyographic activity in limb muscles (Anderson and Behm, 2005). Also, enhanced muscle co-contraction is evident when the landing surface becomes unstable (Shultz et al., 2015). Additionally, when individuals landed on unexpected vs. expected surface, both pre- and post-contact muscle activation as well as activation latency tended to increase (Simpson et al., 2019) (Konishi et al., 2020). These data suggest that unexpected perturbations during landing may immediately facilitate both feed-forward and feed-back neuromuscular control, leading to acute adaptations in terms of improved balance and postural control. Balance perturbation exercises are typical forms of training that target muscle proprioception by forcing the practitioner into unstable postural positions, in which the visuo-motor function is less involved. Perturbations can be evoked by either exercising (Escamilla et al., 2016; Laudner and Koschnitzky, 2010; Lee et al., 2018) or landing (Sebesi et al., 2021; Shultz et al., 2015) on unstable surfaces such as Bosu, Togu, and Dynair pads or Swiss ball. Though the use of these unstable surfaces provides unaccustomed proprioceptive stimuli, they are inadequate for contrasting the effects of expected versus unexpected stimuli.

Taken together, in the present study we tested the hypothesis that the random compared to blocked organization of lower-extremity proprioceptive motor practice, will result in better balance and postural adjustments. To this aim, healthy young adults practiced single-leg drops on a custom-built spring board, which tilted upon landing either into the same directions (blocked condition) or into variable and unknown directions (random condition). To ensure group homogeneity, our investigation was limited to males for various reasons. First, males and females apply different static balancing strategies in terms of postural sway velocity (Andreeva et al., 2020). Females also initiate different lower extremity mechanics during landings than that of males (Salci et al., 2004). Finally, trained collegiate student males represented weaker balance skills then females, quantified with the stability index (Sekulic et al., 2013), suggesting perhaps greater sensitivity to proprioceptive training in males.

## Methods

### Participants

Participants were recruited by posting flyers on university community boards, advertising the study on social media, and by announcing the study at the start of lectures. An a-priori sample size calculation (using G*Power 3.1.9.7, targeting a medium effect size: partial eta squared = 0.06, and a power of 0.80) revealed that 12 participants were necessary for the study. Finally, fifteen healthy male physical education students were included in the present study (mean ± SD; age = 23.4 ± 2.2 years, body weight = 76.8 ± 4.2 kg, height = 182.1 ± 7.8 cm). Inclusion criteria were: experience in the use of unstable surfaces in sport training. Exclusion criteria were: current lower extremity injuries, vestibular abnormalities, and current participation in balance training. Participants’ physical activity level was determined by a self-report questionnaire. In addition to the regular curricular sport courses (2.3±1.4 h/week), participants also pursued additional sport activities (4.0 ± 2.6 h/week). Before the start of measurements, participants received information about the experimental protocol and the potential risks involved in the study and gave written consent based on the Declaration of Helsinki. The University Ethics Committee approved the study protocol and the consent form (7961-PTE2019).

### Experimental Design

**FIGURE 1:**
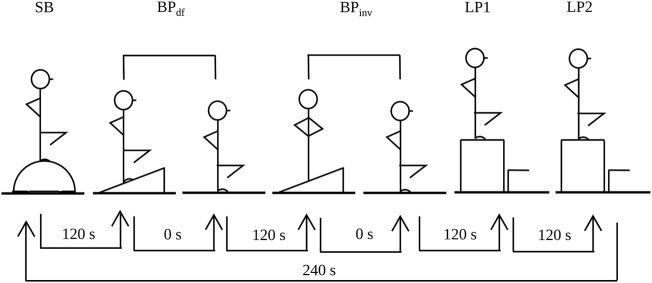
Experimental protocol to compare the acute effects of single leg landings on balance board the top of which tilted into randomized vs. blocked directions on static balance and postural adjustment. SB, static balance test, BP_df_, balance perturbation test in ankle dorsiflexion, BP_inv_, balance perturbation test in ankle inversion, LP, landing practice (either random or blocked).

One week before the beginning of the experiment, participants visited in the laboratory to get familiarized with the performance tests and the proprioceptive training tasks. The experiment itself consisted of two intervention sessions, separated by 1 week. Both intervention sessions involved an acute bout of proprioceptive training and the performance measurements. A within-subject design was used, i.e., participants performed the performance tests and the proprioceptive training (either randomized or blocked) with one leg in intervention session 1 and with the other leg in intervention session 2. The sequence of the training conditions (random, blocked) was counterbalanced across sessions. Leg dominance was also counterbalanced as much as possible, so that eight participants performed the random condition with the dominant leg, and four participants did it first in the sequence, and four participants did it last. Seven participants did the random condition with the non-dominant leg, out of which four participants did it first and three participants did it last. We used the ball kicking task to determine leg dominance. Performance tests (static balance and balance perturbation) were performed before and after proprioceptive training in both intervention sessions. In all sessions, participants warmed-up by riding a stationary bicycle ergometer for 5 min followed by stretching, focusing on lower extremity muscles. Figure 1 shows the experimental protocol and the timeline.

### Static Balance Test

For testing static balance, we followed the methodology by Laudner and Koschnitzky (2010). Participants stood on the bladder (convex) surface of a Togu^®^ Jumper (Togu^®^ GmbH, Prien-Bachham, Germany) with one leg and maintained balance for 10 s. The other knee was flexed to 90° and hands were kept on the hips. During balancing, participants focused on a marker placed on the wall 1.5 m far at eye level. This task was carried out twice, and a 1 min rest was allowed between the trails. Lower extremity kinematic data were collected during the entire 10 s-long balancing.

### Balance Perturbation Test

For balance perturbation, we used an inclined board with the top of 20° angle with respect to the horizontal, which fits within the inclination range used by others (Lin and Nussbaum, 2012). The test consisted of two balance positions: first, participants accommodated to the inclined surface by standing and balancing on the board with one leg with dorsiflexed ankle (toes-up position), for 1 min. Immediately after, participants stepped on the flat surface and maintained balance for an additional 10 s, while kinematic data were collected during the first 5 s. Two minutes later, the same procedure was repeated but participants accommodated in ankle inversion position (Lin and Nussbaum, 2012). Participants were asked to shift from inclined to flat surface with no delay and without looking down on the floor. We used this test to measure the magnitude of the postural after-effects of accommodating to tilt a surface (Kluzik et al., 2005).

### Kinematic Data Processing

**FIGURE 2:**
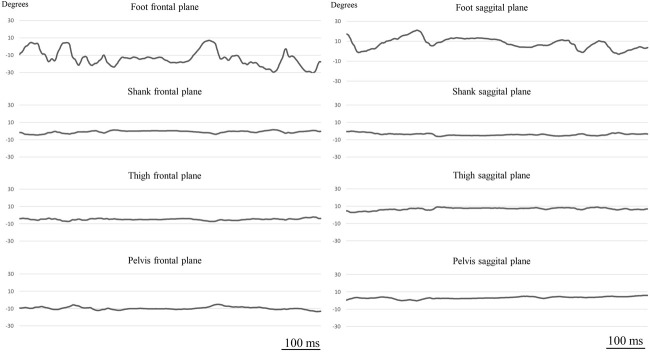
A representation of segmental (foot, shank, thigh, pelvis) orientation angle—time curve (sampling frequency: 100 Hz) obtained in frontal and sagittal planes during balancing on the Togu^®^ Jumper^®^.

During the static balance test and the balance perturbation test, balance performance was quantified using the segmental sway approach (Curtis et al., 2015): the foot, the shank, and the thigh of the stance leg as well as the pelvis was equipped with 3D motion tracking sensors (Noraxon U.S.A. Inc., Scottsdale, United States). The sensors include three orthogonally mounted gyroscopes to sense 3D angular motions. Thus, we were able to determine angular deviation from the vertical axis (orientation angle) in the selected plane. The vertical axis was considered 0°, which was calibrated by requesting the participant to stand still on two legs in upright position for 10 s. We followed the manufacturer’s recommendations concerning sensor positioning and calibration. Briefly, sensors were affixed to the foot: upper foot, slightly below the ankle; for shank: on the tibia bone; for thigh: on the lower quadrant of quadriceps, slightly above the knee cap, and area of lowest muscle displacement in motion; pelvis: on the bony area of sacrum. During both tests, the segmental orientation angle data were recorded with respect to time (Figure 2) at 100 Hz sampling frequency in both the frontal and the sagittal plane, using MyoMOTION hardware (Noraxon U.S.A. Inc., Scottsdale, United States).

For the static balance test (performed on the Togu^®^ Jumper^®^), the length of the orientation angle—time curve was calculated in the entire 10 s-long test period, using the Pythagorean theorem as follows:

$$\sum_{k}^{n}\sqrt{\left({\left[\alpha \left(k\right)-\alpha \left(k-1\right)\right]}^{2}+0.0001\right)}$$

where *α* = segmental orientation angle (°) with respect to the vertical axis (0°), and *k* represents the actual data point on the angle—time curve. The value of 0.0001 was obtained by squaring the 0.01 s sampling interval (at 100 Hz sampling frequency) on the horizontal axis of the angle—time curve. Using this data processing, shorter orientation angle—time curve demonstrated less segmental angular sway and better balance performance.

In the balance perturbation test (accommodation to a tilt surface), the segmental orientation angle values (except for foot) both in the frontal and sagittal plane were recorded and averaged in the first 5 s, after participants stepped off the tilted surface and stood on the flat surface. Any angle deviation from the vertical axis (0°), discounting direction, was defined as absolute error:

Absolute error = │X(°)_participant_ - 0°│,For constant error, the difference between the participant’s segmental angle and zero was used, considering the direction of the error:

Constant error = X(°)_participant_ - 0°.

We adopted the above data processing methodology from the study by Zhang et al. (2019), who quantified joint position accuracy.

### Proprioceptive Training: Random and Blocked Landings on Tilt Surface

**FIGURE 3:**
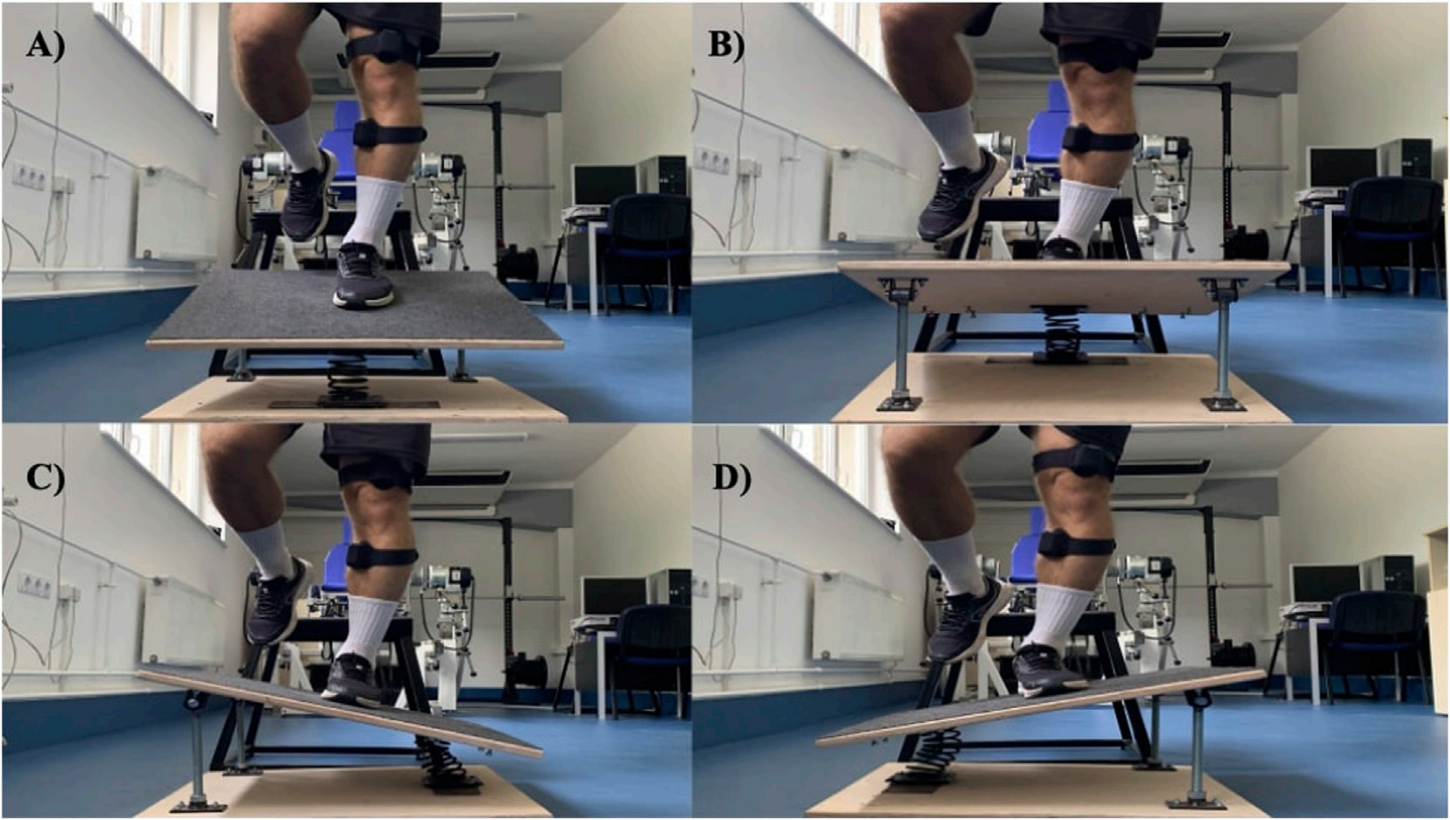
Landings on the custom-built balance board with four different tilt options: **(A)** ankle plantarflexion, **(B)** ankle dorsiflexion, **(C)** ankle inversion, **(D)** ankle eversion.

Figure 3 shows the experimental setup. Participants stood on a 50 cm tall metal stool. The front edge of the stool was 40 cm from the top of the springboard. The springboard consisted of two, 2 cm thick, square-shaped, 0.5 m × 0.5 m hardwood planks. The bottom board never moved and top board could tilt. On one side, the two boards were interconnected with a steel spring (coil diameter of 10 cm). On the other side opposite to the spring, the two boards were interconnected with metal hinges. In the starting position, the two boards were parallel with each other with the spring uncoiled and the hinge straight, setting the two boards 30 cm apart. The spring was compliant enough to allow the top board to tilt when the participant landed on the board on the spring side of the board. The top board was covered with a solid color felt material so that participants were unable to identify its tilt direction before landing. The tilt of the board was set to a given position as the participant stood on the stool with eyes closed. Upon instruction, participant opened his eyes and performed the drop on the board set to a given tilt position. The tilt of the board was set to 20° and the board was manually rotated between trials to provide the four tilt positions during landing.

Participants performed two sets of eight drops and landings with one leg with hands on the hips. After landing, they maintained the landing position for 3 s. There were four tilt positions: dorsiflexion (DF), plantarflexion (PF), eversion (EV), inversion (IN). In the random practice condition, the sequence of tilt directions was randomized by drawing from a hat (Set 1: DF, IN, EV, IN, PF, PF, EV, DF; Set 2: DF, EV, PF, PF, IN, EV, IN, DF). After each trial, one researcher manually rotated the spring board into the next direction, while participants closed their eyes but then participants opened their eyes as they started the landing movement. Each participant performed this same random sequence in the random condition. In the blocked condition, the direction of the tilt was reported to the participants: Set 1: PF, PF, PF, PF, DF, DF, DF, DF and Set 2: IN, IN, IN, IN, EV, EV, EV, EV. To determine total volume/session for our proprioceptive protocol, we extensively studied previous methodologies on the use of unstable surfaces in warm-up procedures in young adults. Differently from our proprioceptive tasks (landings), most studies used static, predominantly standing balancing tasks the volume of which was expressed in durations ranging from 1 min (Gogte, 2017) to as long as 18–25 min (Muehlbauer et al., 2021) (Romero-Franco et al., 2013), the latter inducing significant fatigue. The closest to our methodology was the one reported by Kyranoudis et al. (2020). Accordingly, male soccer players performed 10 repetitions of leg raises and 12 repetitions of single-leg squats per leg on a BOSU^®^ balance trainer, as part of their warm up. We were unable to use studies, in which landings were performed on unstable surface, as they investigated either the effect of their long-term use or the biomechanical mechanisms. Considering the difficulty and the fact that fatigue may increase injury risk in our designated landing task, we arrived to 16 repetitions of landings in both conditions.

### Statistical Analyses

Means and standard deviations were computed for the measured and calculated variables. All data were checked for normality (Shapiro-Wilk test). Variables that did not show normal distribution were log transferred to obtain normality. To test interactions among condition (random and blocked), body segment (foot, shank, thigh, and pelvis) and time (pre and post), three-way ANOVAs of repeated measures were performed for segmental angular sway (dependent variable), obtained in frontal and sagittal planes during the static balance test. In case of significant main effects, where the independent variables consisted of more than two levels, one-way ANOVA was performed. In any pairwise comparisons, the Bonferroni correction was used. The absolute and constant error (dependent variables) values obtained in the balance perturbation test were analyzed with the same procedure (note that data for foot was not recorded in this test as participants stood on straight rigid surfaces). The post-hoc statistical power values obtained for the ANOVA tests are presented in the results section.

## Results

### Static Balance

The frontal and sagittal plane segmental angular sway values for the random and the blocked conditions are presented in Table 1. The ANOVA tests revealed that for the frontal plane segmental angular sway neither the condition by body segment by time interaction nor interaction of any two factors were significant. However, we found significant time main effect (F_1,15_ = 3.5, *p* = 0.043, statistical power = 0.57, partial η^2^ = 0.022), suggesting that overall segmental angular sway reduced from pre- to post-exercise, regardless of practice condition. The time by body segment interaction approached the level of significance (*p* = 0.072). The body segment main effect was significant (F_1,15_ = 100.8, *p* < 0.0001, statistical power = 0.89, partial η^2^ = 0.12), and the post-hoc test revealed that, except between shank and thigh, the segmental angular sway was significantly different among any segments (*p* < 0.05, Cohen’s *d* value range = 0.29–0.62), regardless of practice condition and test time.

**TABLE 1:** Frontal and sagittal plane segmental angular sway values (mean ± SD) for the random and the blocked conditions obtained during the static balance test (10-s-long single-leg standing on the convex surface of a Togu^®^ Jumper). Values represent angle-time curve length.

	Random				Blocked			
	Pre		Post		Pre		Post	
Frontal plane angular sway (°)								
Pelvis	63	±26	56	±33	64	±46	63	±36
Thigh	56	±14	50	±47	71	±23	67	±76
Shank	55	±13	48	±17	70	±49	68	±44
Foot	397	±97	333	±79	444	±138	395	±102
Sagittal plane angular sway (°)								
Pelvis	42	±15	41	±17	43	±31	42	±15
Thigh	74	±23	69	±34	84	±54	75	±36
Shank	62	±14	57	±16	70	±21	68	±19
Foot	166	±52	138	±48	174	±58	154	±39

In sagittal-plane segmental angular sway, neither the condition by body segment by time interaction nor interaction of any two factors were significant. The time main effect only approached the level of significance (F_1,15_ = 3.3, *p* = 0.069). The body segment main effect was significant (F_1,15_ = 104.1, *p* < 0.0001, statistical power = 0.93, partial η^2^ = 0.09), and the post-hoc tests revealed that, except between shank and thigh, the segmental angular sway was significantly different among any segments (*p* < 0.05, Cohen’s *d* value range = 0.35–0.67).

### Postural Adjustment

The absolute and constant error values obtained for each body segment and each plane are presented in Table 2. In the frontal plane, we found neither significant time main effects nor significant interactions among any independent variables either for absolute or for constant error.

**TABLE 2:** Absolute (ignoring directions) and constant (considering directions) error values obtained by quantifying angular deviations from the vertical axis for each segment during the balance perturbation tests. Frontal and sagittal plane values represent angular deviations obtained when participants shifted form tilt surface (ankle inversion and ankle dorsiflexion, respectively) to flat surface.

	Random				Blocked			
	Pre		Post		Pre		Post	
Absolute error (°)								
Frontal plane								
Pelvis	3.0	±2.5	3.2	±2.7	4.2	±3.4	3.6	±3.2
Thigh	2.6	±1.7	2.4	±1.5	2.6	±1.0	3.0	±2.1
Shank	2.9	±1.0	2.8	±1.0	3.1	±1.5	3.2	±1.4
Sagittal plane								
Pelvis	3.5	±2.7	2.3	±1.6	3.9	±3.2	3.4	±2.0
Thigh	2.7	±1.7	2.8	±1.6	4.0	±3.1	3.9	±3.6
Shank	3.5	±2.8	2.7	±2.4	4.0	±3.3	2.4	±2.7
Constant error (°)								
Frontal plane								
Pelvis	1.8	±3.5	1.6	±3.9	2.9	±4.6	2.2	±4.4
Thigh	0.7	±3.1	0.6	±2.8	0.2	±2.9	1.0	±3.6
Shank	0.7	±3.1	0.9	±2.9	0.9	±3.4	1.3	±3.4
Sagittal plane								
Pelvis	3.0	±2.6	3.3	±2.7	3.3	±2.3	3.0	±2.7
Thigh	2.6	1.8±	2.4	1.6±	3.2	±1.5	3.3	1.4±
Shank	2.9	±1.1	2.8	±1.0	3.2	±2.6	3.3	±2.7

In the sagittal plane, for the absolute error, we found no significant interactions among any independent variables, however, the time main effect was significant (F_1,11_ = 3.1, *p* = 0.047, statistical power = 0.48, partial η^2^ = 0.017). For constant error, neither the time main effect nor any interactions were significant.

## Discussion

An important limitation in previous studies examining the effects of random vs. blocked practice on motor performance was the use of intervention tasks that require pro-active actions processed by vision (Afsanepurak et al., 2012; Gaspar et al., 2019; Porter and Magill, 2010). However, sudden balance perturbations are processed reactively and not through visually-aided actions (Quant et al., 2004). In the present study, we applied a unique intervention and compared the acute effects of lower-extremity proprioceptive stimuli organized into either a randomized or a blocked sequence. The data revealed that a single-session practice of landings on a tilt surface induced favorable changes in both static balance and postural adjustment, but these improvements were independent of practice conditions, rejecting the hypothesis.

A methodological novelty in the present study is that we organized the same proprioceptive training stimuli into either a randomized or a blocked practice order, while controlling for other training variables that may affect practice effects. Several studies used unstable surfaces (balance pad, TOGU^®^, BOSU^®^, wobble board, Airex^®^, Swiss ball) in interventions to improve proprioception and balance. Either standing or exercising on these surfaces would force practitioners to lose balance which would in turn evoke proprioceptively-mediated reflexes. The direction and the nature of balance losses are, however, variable and uncontrolled by researchers when typically two or more unstable surfaces of different shapes and materials or unstable surfaces with flat surface are compared in interventions to investigate their differential effects on postural sway (Bouillon et al., 2019; Lee et al., 2018; Lubetzky-Vilnai et al., 2015; Nepocatych et al., 2016; Strøm et al., 2016; Zemková, 2017). With the springboard and the methodology developed in the present study, we were able to compare unexpected randomized proprioceptive stimuli with expected blocked stimuli, while ensuring that not only practice volume (number of repetitions and sets) but also tilt angles and number of tilt directions be identical in the two conditions.

Acute adaptation to the practice in the present study was tested by measuring static balance and postural adjustment after balance perturbation. A lack of condition by time interactions in all measured variables suggests that improvements were uniform after random and blocked practice, rejecting our hypothesis. The time main effect in angular sway was significant in frontal-plane, and nearly significant in sagittal-plane, suggesting overall reductions in static balance sway. Static balance is considered an important skill not only in athletic performance but also in the prevention of falls and injuries, and it is often tested after long-term proprioceptive training (see review by Hrysomallis, 2007). Moreover, a single session balance training is often performed by athletes as part of their warm-up to acutely enhance performance and prevent injuries in the subsequent physical activity (Muehlbauer et al., 2021). Previously, it was demonstrated that a single session of unilateral standing on a balance board can immediately reduce postural sway (Marcori et al., 2020; Muehlbauer et al., 2021). These studies, however, applied volumes as large as a total of 540–600 s unilateral balancing, performed within 30 min. The significance of our result is that as few as 16 unilateral drops and landings on a tilted surface improved static balance performance as shown by the reduced overall lower extremity postural sway in the frontal plane and by the non-significant trend in sagittal plane.

The segmental sway approach allowed us to quantify lower-extremity segment-specific adaptations in static balance, and the time-body segment interaction was nearly significant in the frontal plane as foot and pelvis angular sways tended to reduce the most and the least, respectively (14% vs. 4%). The preferential ankle joint stabilization after the intervention can be attributed to two factors. First, the landings on the tilt surface applied in the intervention provoked quick and large range of motion changes in the ankle joint, enhancing greater adaptation. Second, the static balance test itself was found to be the most sensitive to foot sway, as revealed by the post-hoc test after significant body segment main effect obtained in both frontal and sagittal planes. Therefore, the present study provides important data on the segment-specific involvement during the static balance test performed on a Togu® Jumper. The sagittal-plane time-body segment interaction was not significant in the static balance test, probably because the foot can be more stabilized by the stronger plantar- and dorsiflexor muscles vs. evertor and invertor muscles.

The balance perturbation test was used to quantify the magnitude of postural distortion after stepping off an inclined surface. The idea is that as surface changes orientation, there is a change in the gravity reference and the support surface reference for postural control (Kluzik et al., 2007). Previous research has extensively investigated the biomechanical mechanisms of the immediate after-effects of surface inclination (Kluzik et al., 2005; Mezzarane and Kohn, 2007; Sasagawa et al., 2009), but the exercise-induced changes are unclear. Our results show that a single session of landings on the springboard improved overall shank-thigh-pelvis alignment perturbed by the tilt surface, although, this improvement was limited to sagittal plane (tested in the toe-up position) and was independent of body segment and practice condition (as shown by the significant time main effect obtained for absolute error).

It is interesting that, even though our intervention involved lateral tilts, the frontal-plane alignment measured after ankle inversion perturbation did not improve. The concept of using such lateral perturbation is that participants are accustomed to asymmetric weight bearing, challenging medio-lateral balancing (Lin and Nussbaum, 2012), but the lack of improvement remains unclear in our study. The functional characteristics of the musculoskeletal system could be an explanation: specifically, in sagittal plane, posture can be efficiently modified by ankle, knee, and hip joint extensions-flexions as well as trunk and head forward-backward positioning. In frontal plane, however, knee joint movement is limited, probably affecting upper segments as well, therefore, our participants may have compensated with trunk and head lateral flexion to maintain balance after perturbation, the segments from which we have not recorded angular data.

Despite that the sagittal-plane absolute error reduced in the balance perturbation test, the constant error remained unchanged. The constant error provides information about the direction to which the given segments’ position is deviated from the vertical axis. Our data therefore suggest that though shank-thigh-pelvis alignment improved, our participants applied variable postural adjustment strategies with their lower extremity.

Though the segmental approach in testing segmental sways and postural adjustment after proprioceptive training provides useful and important findings in the present study, we did not measure head and trunk sway. Future studies should examine total-body segmental involvement in practice-induced adaptation, especially when surface perturbation is performed laterally in the perturbation test. Another important limitation is that we did not measure how long practice effects lasted, though performing the tests more frequently within a session would probably evoke significant practice effects. Finally, blocks of four repetition landings with the same tilt directions in the blocked practice condition was probably too few to produce a big enough differential effect compared with the effects produced by random practice. The effects of long-term intervention, therefore, with larger volume and with repetitions organized into larger blocks in the blocked condition should be studied. In conclusion, results of the present study suggest no differences in the short-term effects of randomized vs. blocked proprioceptive practice on healthy young adults’ balance and postural control. However, the improvements were test- and plane-specific. Overall, lower extremity postural sway during static balancing reduced both in the frontal and the sagittal planes, the ankle sway in a greater extent. Postural adjustment improved only in the sagittal plane, and improvements were achieved by individual adjustment strategies.

## Data Availability

The original contributions presented in the study are included in the article/Supplementary Material, further inquiries can be directed to the corresponding author.
